# Studentized continuous wavelet transform (*t*-CWT) in the analysis of individual ERPs: real and simulated EEG data

**DOI:** 10.3389/fnins.2014.00279

**Published:** 2014-09-10

**Authors:** Ruben G. L. Real, Boris Kotchoubey, Andrea Kübler

**Affiliations:** ^1^Department of Psychology I, Institute of Psychology, University of WürzburgWürzburg, Germany; ^2^Institute for Medical Psychology and Behavioural Neurobiology, University of TübingenTübingen, Germany

**Keywords:** electroencephalogram, EEG, wavelet, *t*-CWT, ERP, significance, detection

## Abstract

This study aimed at evaluating the performance of the Studentized Continuous Wavelet Transform (*t*-CWT) as a method for the extraction and assessment of event-related brain potentials (ERP) in data from a single subject. Sensitivity, specificity, positive (PPV) and negative predictive values (NPV) of the *t*-CWT were assessed and compared to a variety of competing procedures using simulated EEG data at six low signal-to-noise ratios. Results show that the *t*-CWT combines high sensitivity and specificity with favorable PPV and NPV. Applying the *t*-CWT to authentic EEG data obtained from 14 healthy participants confirmed its high sensitivity. The *t*-CWT may thus be well suited for the assessment of weak ERPs in single-subject settings.

## 1. Introduction

A wide variety of traumatic and non-traumatic brain injuries can lead to disorders of consciousness (DOC), the vegetative state (aka. apallic syndrome) and the minimally conscious state being the most severe forms (Laureys et al., 2006). Event-related potentials (ERPs) promise to objectively assess residual cognitive functions in these patients (Kotchoubey et al., 2002, 2005; Kübler and Kotchoubey, 2007; Monti, 2012). However, several factors have been noted which make the reliable assessment of ERPs in these patients challenging: EEG recorded at the patients bed-side is often contaminated by artifacts from the surrounding medical equipment or sudden changes in the patient's sympathetic activity, e.g., excessive sweating, changes in body temperature, blood pressure, heart and respiratory rate, or body posture. Further, increasing the number of trials, a method often used to increase the signal-to-noise ratio (SNR), is limited by the rapidly fluctuating vigilance and the short attention span of these patients (Neumann and Kotchoubey, 2004; Laureys et al., 2006). These issues are all the more important, since neuroscientific findings of preserved cognitive functioning in DOC patients may influence the patient's further medical treatment (Laureys et al., 2006), or questions concerning end-of-life decisions (Eisenberg, 2008).

Thus, any EEG analysis technique should fulfill at least four requirements. Firstly, to maintain reliability and, thus, validity, it should be independent of the experimenter's expertise (Valdes-Sosa et al., 1987). Secondly, it should allow for the statistical evaluation of identified ERPs. Thus, the technique must be applicable to single subject analysis and, therefore, must use single trials for statistical evaluation. Thirdly, the technique should be able to differentiate temporarily distinct ERPs (Bostanov and Kotchoubey, 2006). Finally, it should show high sensitivity, i.e., correctly identifying those subjects showing the ERP of interest, and high specificity, correctly identifying those subjects who do not show the ERP of interest.

It should be noted that here the term ERP is used in a mathematical/statistical sense, i.e., it means a time-locked deflection which discriminates between two experimental conditions or between one experimental condition and baseline activity. This definition makes no assumption as to the underlying physiological or psychological generators.

In this paper, we describe an ERP detection method based on the continuous wavelet transform (CWT), and compare its performance to a variety of competing analysis techniques in detecting ERP components in artificial and authentic EEG data under varying SNRs.

## 2. Methods

### 2.1. The studentized continuous wavelet transform (*t*-CWT)

**Figure 1:**
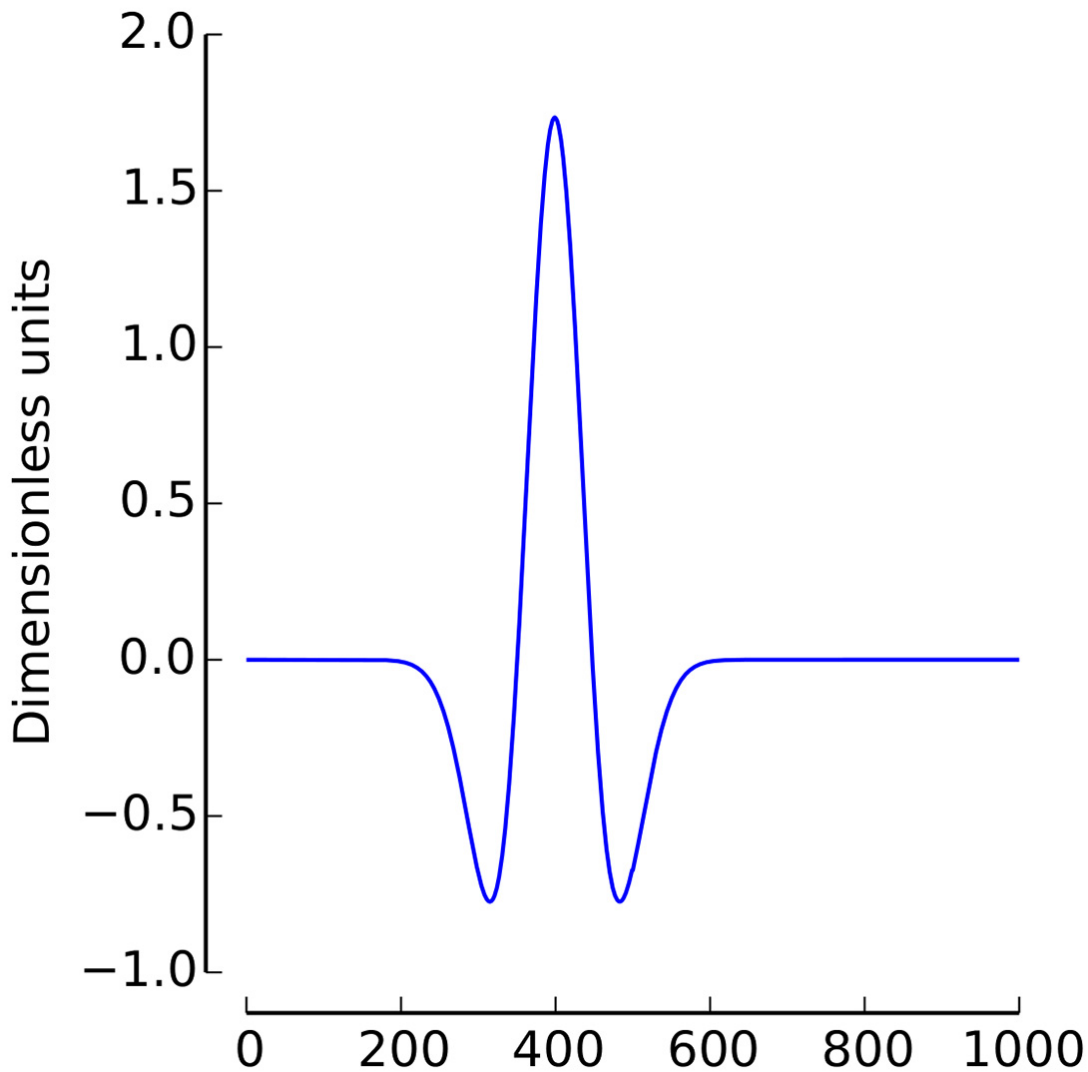
**A Mexican Hat Wavelet with a scale of 200 and a time shift of 400 ms**.

**Figure 2:**
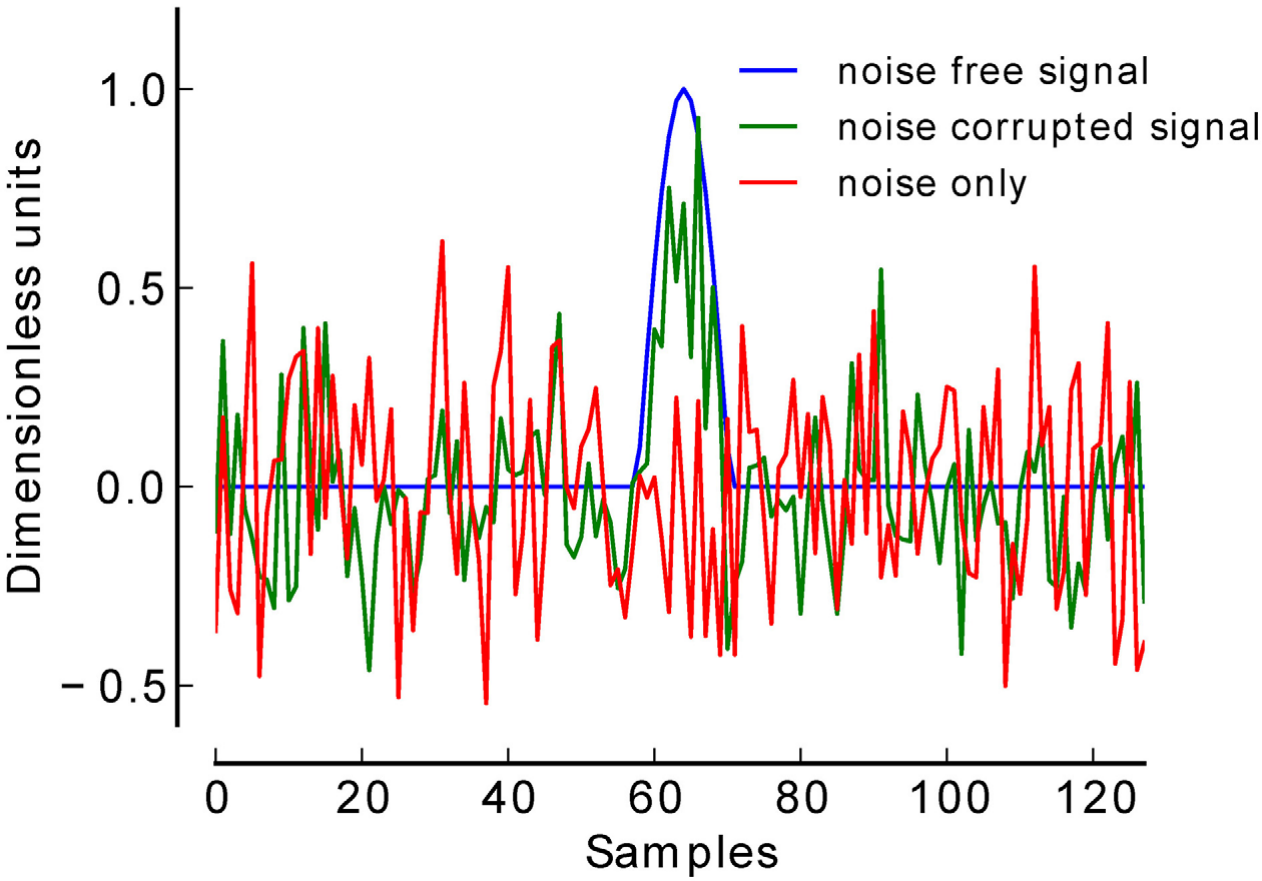
**Average of simulated EEG data at −16 dB**.

**Figure 3:**
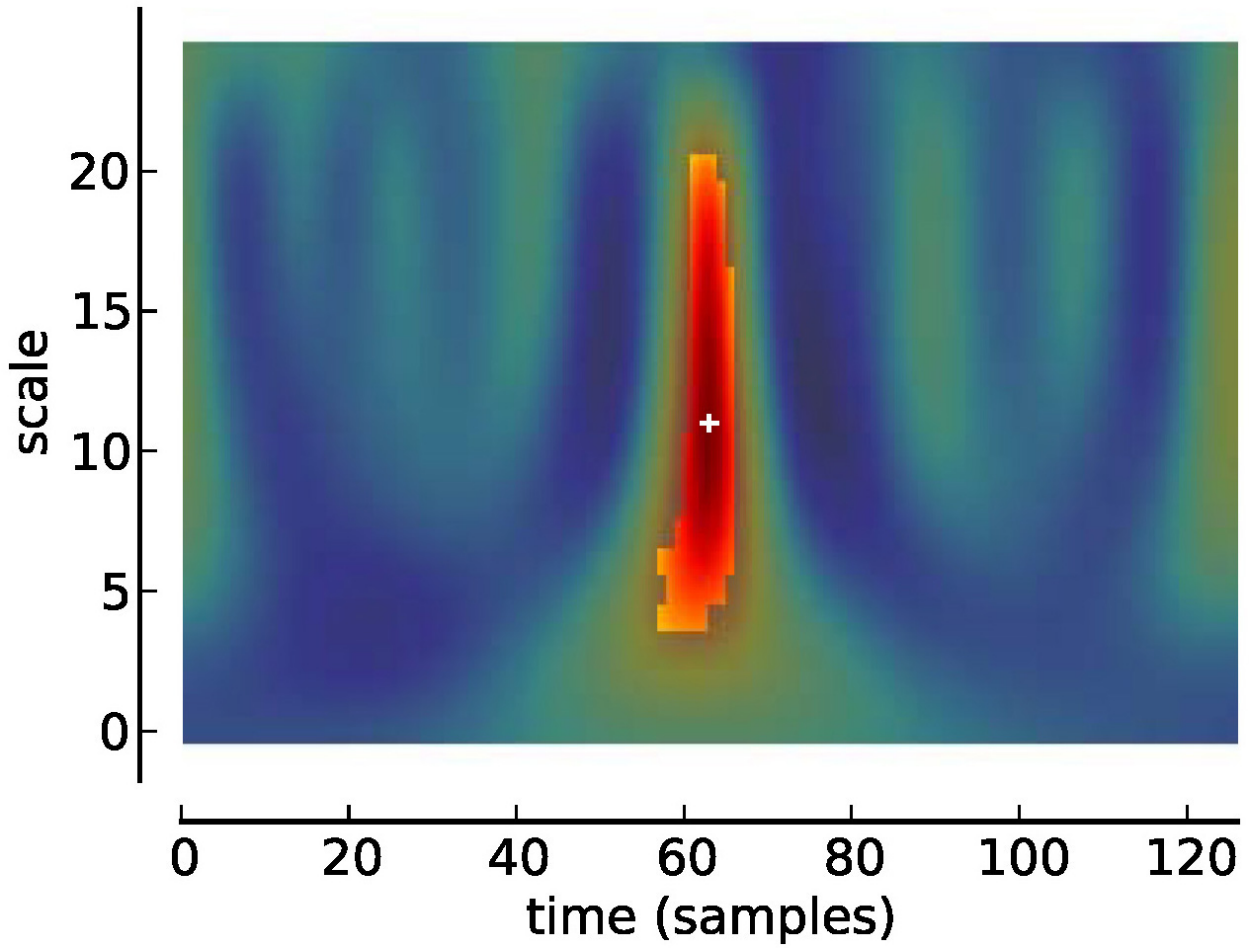
**Scalogram of Studentized wavelet coefficients corresponding to Figure 2**. Highlighted area indicates location of significant (*p* < 0.05) differences. Plus sign indicates local maximum.

Classical techniques for ERP detection in data obtained from a single subject include template matching (Woody, 1967) or peak picking after low-pass filtering (Ruchkin and Glaser, 1978), which, incidentally, are virtually the same, since low-pass filtering can be thought of as determining the cross-covariance of a signal with a predefined template. Later, the discrete wavelet transform (DWT) has been suggested (Samar et al., 1999). While DWTs provide for a very economical signal representation, the resulting coefficients are difficult to interpret in terms of the characteristics of an ERP: Typically, one ERP is reflected in several coefficients and, conversely, one coefficient may also reflect several ERPs. Further, in ERP assessment, complete representation of a signal is a minor requirement in comparison to the overall aim of extracting meaningful features from the data. Thus, our approach concentrates on feature extraction and uses the continuous wavelet transform (CWT) to represent EEG signals as a function of two parameters: time and scale. The resulting coefficients can be thought of forming a map with the axes corresponding to time and scale—a scalogram (see Figures 1–3). In this map, local extrema indicate salient features of the EEG signal, such as peaks or oscillations.

In the following, we describe and evaluate a variant of the Studentized Continuous Wavelet Transform (*t*-CWT), in which Student *t*-values are calculated for each wavelet coefficient (Bostanov and Kotchoubey, 2006) and evaluated using a *t*_max_ randomization test (Blair and Karniski, 1993; Groppe et al., 2011). Previous implementations of the *t*-CWT (Bostanov, 2004; Bostanov and Kotchoubey, 2006) included a time-dependent low-pass filtering procedure, which attenuated short deflections occurring late in an epoch. This procedure was originally implemented to account for the phenomenon that earlier ERPs are shorter than late ERPs and that short deflections occurring late in an epoch are less unlikely to represent a true ERP. However, this procedure makes rather strict assumptions on the distribution of ERP components, thus running the risk of attenuating ERPs, which do not match the filter specifications. In data from healthy participants this assumption may be less critical than in patients with acquired brain damage, who often exhibit substantial variation in latencies. For example, analyzing EEG data obtained from patients with severe disorders of consciousness, Guérit et al. (1999) found that latencies of a P300-like component ranged from 260 to more than 620 ms after stimulus onset. With this in mind, we chose not to implement the time-dependent low pass filtering procedure thus avoiding the risk of attenuating ERPs outside the filter's specification.

*First step:* Calculation of the Continuous Wavelet Transform (Mallat, 2007)

For a digitally sampled EEG signal *f*^*mo*^[*t*] of length N, where m denotes the channel, o denotes the trial, and t denotes the time variable, the wavelet coefficients *W*^*mo*^[*s*, τ] are calculated as follows:

(1) $$W^{mo}[s,\;\tau ]=\frac{1}{\sqrt{s}}\;\sum_{t\;=\;0}^{t\;=\;N-1}f^{mo}[t]\psi \left(\frac{t-\tau }{s}\right)$$

where τ denotes the time shift and *s* > 0 denotes the scale. Both τ and *s* are measures in time units, and ψ is the wavelet function.

(2) $$-\psi (\tau )=\frac{2}{\pi^{1/4}\sqrt{3\sigma }}\;\left(1-\frac{\tau^{2}}{\sigma^{2}}\right)\;\exp\;\left(\frac{-\tau^{2}}{2\sigma^{2}}\right)$$

In the current study, the Mexican Hat wavelet was used (2, with σ = 1/4, see Figure 1)^1^.

Equation (1) implies that the CWT's representation of a signal is highly redundant. While this redundancy is not very efficient, i.e., the CWT generates many more coefficients than the DWT, it does allow for the precise localization of ERPs.

*Second step:* Calculation of Student *t*-values

In standard ERP analysis, comparison of within-condition averages is often used to decide where activation differs between experimental conditions. The same logic is followed here, with the exception that Student *t*-values are calculated instead of means. In the two-sample case, as when comparing two experimental conditions, *t*-values are calculated using the two-sample *t*-test. In the one-sample case, when comparing activity against the baseline, a one-sample *t*-test can be used. The result of this procedure is a statistical map, which shows the reliability of each wavelet coefficient across trials. However, the *t*-values are not directly used for statistical analysis. The primary reason is that the statistical map corresponds to many individual *t*-test, thereby introducing the problem of multiple comparison. Another reason is that the distribution of Studentized wavelet coefficients is unknown, rendering the statistical validity of parametric statistical tests questionable. The solution to this problem is addressed in Section 2.2.

*Third step:* Detection of local extrema

From the statistical map local extrema (*s*^*mi*^, τ^*mi*^) are detected. These are the locations in the time-frequency plane of locally maximal differences (weighted by variance) between experimental conditions. Note that no weighting procedure is used in the detection of local extrema. Spurious local extrema—which do not correspond to true differences in activity in the data—are deleted during significance testing (see Fourth Step).

*Fourth step:* Calculation of significance

The purpose of this step is to ascertain whether the local extrema identified in the previous step truly differentiate between the two experimental conditions. The wavelet coefficients calculated in the First step are subjected to a randomization test, described in the next section. After randomization testing, a *p*-value can be assigned to each local extremum indicating significance.

### 2.2. Randomization tests

Two-sample tests are designed to assess whether a statistic differs between two experimental conditions. Under the null hypothesis of no difference, the information that an observation originated from a particular condition is quite meaningless, since the same observation could just as likely have originated from the other condition, i.e., the condition labels assigned to each observation are *exchangeable*. Under the null hypothesis, then, the significance of a statistic expressing the group difference, such as a two-sample *t*-value, can be assessed by comparing the original statistic with the distribution of this statistic obtained when the condition labels have been permuted, or, if permutation is not feasible, when they have been randomly exchanged many times.

Importantly, this procedure can be easily extended to compensate for the increased chance of false positive findings due to increased number of comparisons. As the number of comparisons increases, so does the likelihood of getting extreme observations by chance. By computing the distribution of the most extreme statistic, the maximal *t*-value (*t*_max_) in our study, across the number of tests for each permutation, the distribution obtained through randomization automatically adapts for the increased likelihood of extreme values (Blair and Karniski, 1993; Groppe et al., 2011).

### 2.3. Application to simulated data

Previously, the *t*-CWT was validated on real EEG data from healthy subjects. The underlying assumption is that the most common ERPs should be present in every healthy subject. Then, an analysis method is the better the more subjects it identifies as showing the ERPs of interest (Bostanov and Kotchoubey, 2006). However, it is also known that even highly prototypical ERPs, such as the P300, may be absent in as much as 31% of healthy subjects (e.g., Lulé et al., 2013). Therefore, the number of subjects in which an ERP can be detected may be a spurious criterion. An arguably better criterion may then be the evaluation of sensitivity, i.e., the number of subjects in which an ERP is truly present and detected, and specificity, i.e., the number of subjects in which a component is truly absent and not detected. However, accurate knowledge of the true presence or absence of ERPs is typically not available. In the past, this problem has been approached by using experts' ratings as a “gold” standard against which automated ERP detection procedures could be validated. However, this procedure finds its difficulties in that inter-rater agreement varies between studies (Valdes-Sosa et al., 1987; Wilson et al., 1996), and that this procedure does not allow for the easy analysis of ERP datasets with different signal-to-noise ratios (Schneider et al., 2003). Lastly, obtaining ratings from two or more experts is expensive and time consuming.

However, simulating ERPs allows for precisely controlling presence or absence of defined components. We therefore generated artificial EEG datasets which either contained or did not contain a signal of interest, and compared the performance of the *t*-CWT to a variety of other ERP detection methods.

#### 2.3.1. Generation of artificial EEG signals

The signal-to-noise ratio (SNR) of EEG signals is often very low and subject to considerable heterogeneity. While ERPs of some subjects exhibit exceptionally high SNRs of up to 3 dB, SNRs in around 50% of healthy participants are lower than approximately −9 dB (Coppola et al., 1978).

To validate our procedure, we simulated a total of 12.000 EEG datasets at six low levels of SNR. For each SNR level (−18 to −13 dB) 1000 datasets were generated in which a simulated ERP (centered positive half wave of a 3 Hz cosine wave) (Yeung et al., 2004) was truly present, and 1000 datasets in which this component was truly absent (see Figure 2). Each dataset consisted of 60 trials of simulated EEG data of one-second duration (sampling rate 128 Hz). In datasets belonging to the present condition, 30 trials contained the positive peak, and the remaining 30 trials did not contain such a peak, thus simulating two experimental conditions. Gaussian white noise was added to each trial to achieve the desired SNR. Datasets belonging to the absent condition were pure Gaussian white noise. The number of 30 trials per condition was chosen following reports that in a classical P300 oddball paradigm at least 20 trials are needed to be able to detect a P300 (Cohen and Polich, 1997).

#### 2.3.2. EEG analysis methods

The performance of the *t*-CWT was compared to five other signal processing methods. Given the tremendous amount of effort invested into the development of new signal processing methods, it is clear that our choice of comparison methods is restricted. The *t*-CWT as proposed in this paper is a combination of two factors, wavelet analysis (which may be understood as simultaneous filtering) and the *t*_max_ randomization test to correct for multiple comparisons. Consequently, data filtering and correction for multiple comparisons were also considered in the choice of comparisons methods. A further consideration was the availability of analysis methods, with filtering and peak picking procedures being implemented in all major EEG analysis software packages (e.g., BrainVision Analyzer, Brain Products, Gilching, Germany) and (*t*_max_) randomization testing being freely available as a software package for the EEGLAB suite (Delorme and Makeig, 2004; Groppe et al., 2011). The *t*-CWT was compared to the following procedures:

1. *Simple peak detection:* A difference signal was calculated by subtracting the average of trials which might contain a peak from the average of trials without a peak, and a two-sample *t*-test performed at the location of the maximum difference. It was expected to provide very high sensitivity but very low specificity. Low specificity was hypothesized since this procedure does not control for α error inflation due to multiple comparisons.
2. *Peak detection after band pass filtering:* As above, but here a fourth order Butterworth band pass filter (0.1–20 Hz) was used before calculation of averages. Filtering of EEG data is often used to increase the SNR, so we expected this procedure to increase sensitivity and specificity. However, because no adjustment for multiple comparisons was performed in this analysis, the false positive rate was expected to exceed the nominal α level.
3. *t*_max_ *based peak detection:* A *t*_max_ randomization test was performed on the unfiltered EEG signal, and the minimal *p*-value selected. This method was expected to show high specificity, reflecting effective control of α-error inflation built into the *t*_max_ randomization test, but at the same time low sensitivity, confirming the low levels of SNR in our datasets.
4. *t*_max_ *based peak detection after band pass filtering:* A *t*_max_ randomization test was used on the fourth order Butterworth band pass filtered (0.1–20 Hz) EEG signal. This procedure was expected to show high sensitivity, reflecting the increased SNR after filtering, and high specificity.
5. *Range based peak picking after band pass filtering:* As (2.), but here the means around the detected peak (± 83 ms) were used for statistical analysis. This method resembles a popular approach of visually determining the latency of the ERP of interest and then calculating the mean-amplitude in an interval surrounding the identified peak.
6. *t*-*CWT:* The *t*-CWT was calculated using five steps per octave to generate logarithmically spaced scales between 1 Hz and half the Nyquist frequency (32 Hz). It was expected to show superior performance to all other methods.

One-thousand repetitions (Groppe et al., 2011) were used for randomization testing of the *t*_max_ tests and the *t*-CWT and α = 0.05 was the nominal false positive rate used for all analyses.

#### 2.3.3. Statistical analysis

Statistical analysis was based on the *F*_1_-score, the harmonic mean of the positive predictive value, *PPV* = *TP*/(*TP* + *FP*), and sensitivity, *TP*/(*TP* + *FN*).

(3) $$F_{1}\;=\frac{2\cdot PPV\cdot sensitivity}{PPV+sensitivity}$$

(4) $$=\frac{2\cdot \text{TP}}{2\cdot \text{TP}+\text{FN}+\text{FP}}$$

*F*_1_ scores are a popular metric in research on information retrieval. In this application setting, a “good” algorithm would retrieve not only all relevant documents (i.e., high sensitivity), but at the same time ensure that the proportion of relevant documents is high in relation to the total number of findings (i.e., high PPV).

While the exact distribution of *F*_1_ is unknown, Goutte and Gaussier (2005) have shown that Monte Carlo simulations can be used to estimate the probability that the *F*_1_ scores of one system (*F*^1^_1_) exceed the scores of another system (*F*^2^_1_). This can be achieved by creating large (50.000 in our case) samples ({*f*^1^_*i*_}_*i* = 1 … *L*_ and {*f*^2^_*i*_}_*i* = 1 … *L*_) of the distributions of *F*_1_ scores using random gamma variates.

(5) $$F_{1}=\frac{U}{U+V}\text{ with }\{\begin{array}{ll} U & \sim \Gamma (TP+0.5,2)\\ V & \sim \Gamma (FP+FN+1,1) \end{array}$$

The probability *P*(*F*^1^_1_ > *F*^2^_1_) is then estimated by:

(6) $$\hat{P}(F_{1}^{1}>F_{1}^{2})=\frac{1}{L}\sum_{i\;=\;1}^{L}\text{I}\left(f_{i}^{1}>f_{i}^{2}\right)$$

where the indicator function I(·) is 1 if the condition is true, 0 otherwise.

A potential problem associated with the sole reliance on *F*_1_ scores is that they are insensitive to the number of true negatives (Sokolova and Lapalme, 2009). While this is not a problem in the classic domain of information retrieval, in individual ERP assessment knowledge about the absence of a particular ERP is often important information. Thus, to complement the traditional *F*_1_ score, we defined the “negative” *F*_1_ score as the harmonic mean of the NPV and specificity:

(7) $$\text{negative }F_{1}=\frac{2\;\cdot \text{ TN}}{2\;\cdot \text{ TN}+\text{FP}+\text{FN}}$$

Finally, we also calculated 95% confidence intervals for the *F*_1_ scores. This was performed by first calculating the distribution of *F*_1_ scores according to (4, 7, 5) and then selecting the *F*_1_ scores delimiting the 2.5 to 97.5% interval.

### 2.4. Application to real EEG data

**Figure 4:**
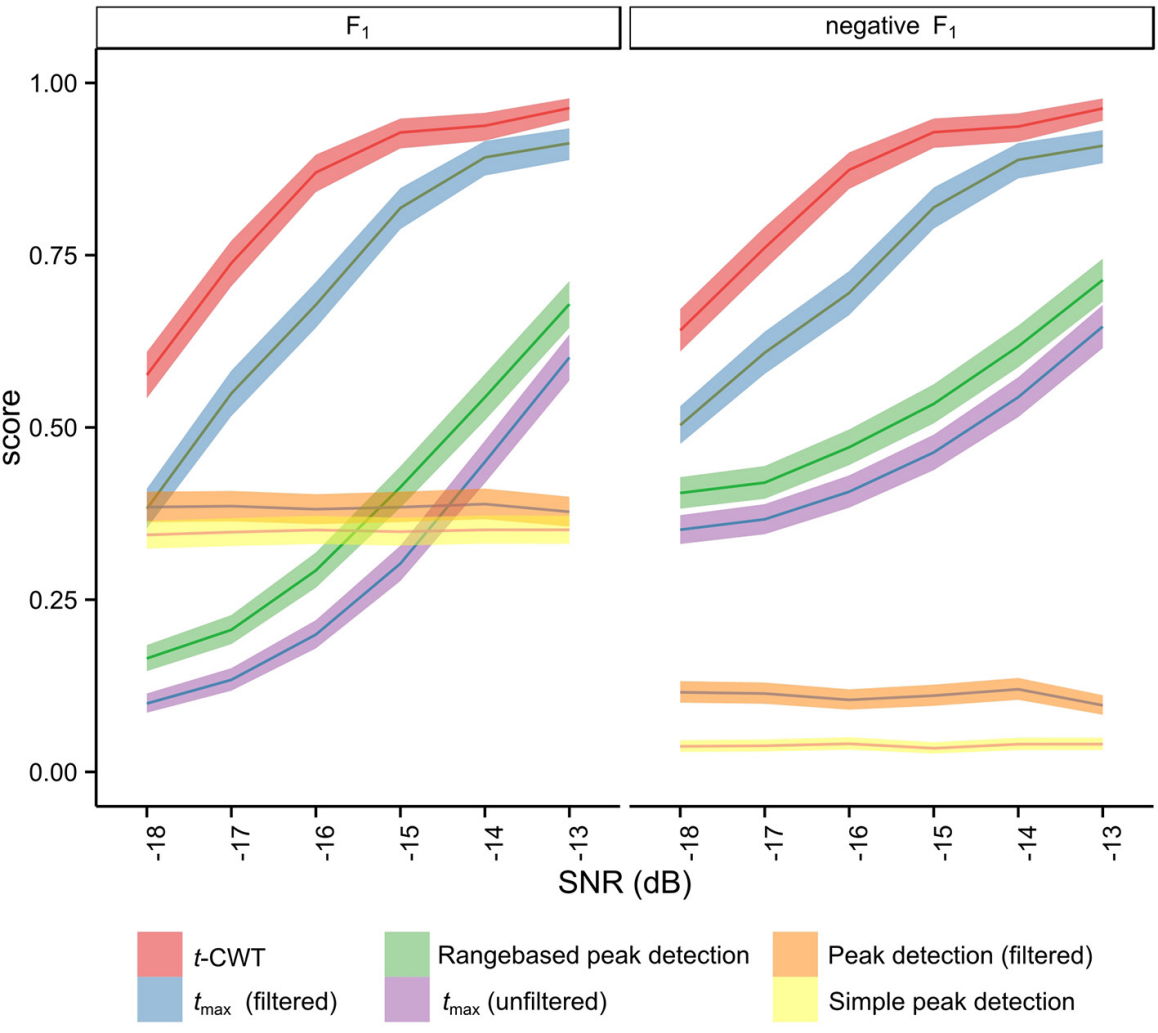
**Means and 95% confidence intervals of the distribution of *F*_1_ after Monte Carlo simulation (5)**.

Anticipating results presented later (see Section 3.1), results from simulation studies indicated an overall favorable performance of the *t*-CWT, closely followed by the *t*_max_ randomization test after band pass filtering (see Figure 4, **Table 2**). To confirm these findings in real EEG datasets, we analyzed EEG data recorded from 14 healthy participants (9 female; mean age = 27.6, *SD* = 9.5) while they listened to a two-tone auditory oddball paradigm. Participants were instructed to silently count the number of odd tones. EEG recordings were performed at the psychophysiological laboratories at the Universities of Tübingen and Würzburg. The study was approved by the local Ethical Review Boards of the institutions involved and conformed to the Declaration of Helsinki (World Medical Association, 2008).

#### 2.4.1. Stimuli

Stimuli were delivered via in-ear headphones (E-A-RTONE Gold, Auditory Systems, Indianapolis, Indiana). Participants were presented with a binaural stream of 420 short complex high (440 + 880 + 1760 Hz) tones into which 60 short complex low (247 + 494 + 988 Hz) tones were pseudo-randomly interspersed (Kotchoubey et al., 2005). Stimulus duration was 50 ms, linear rise-fall time was 5 ms, intensity was 70 dB (Polich, 1986), and SOA was 850 ms.

#### 2.4.2. EEG recording and preprocessing

**Figure 5:**
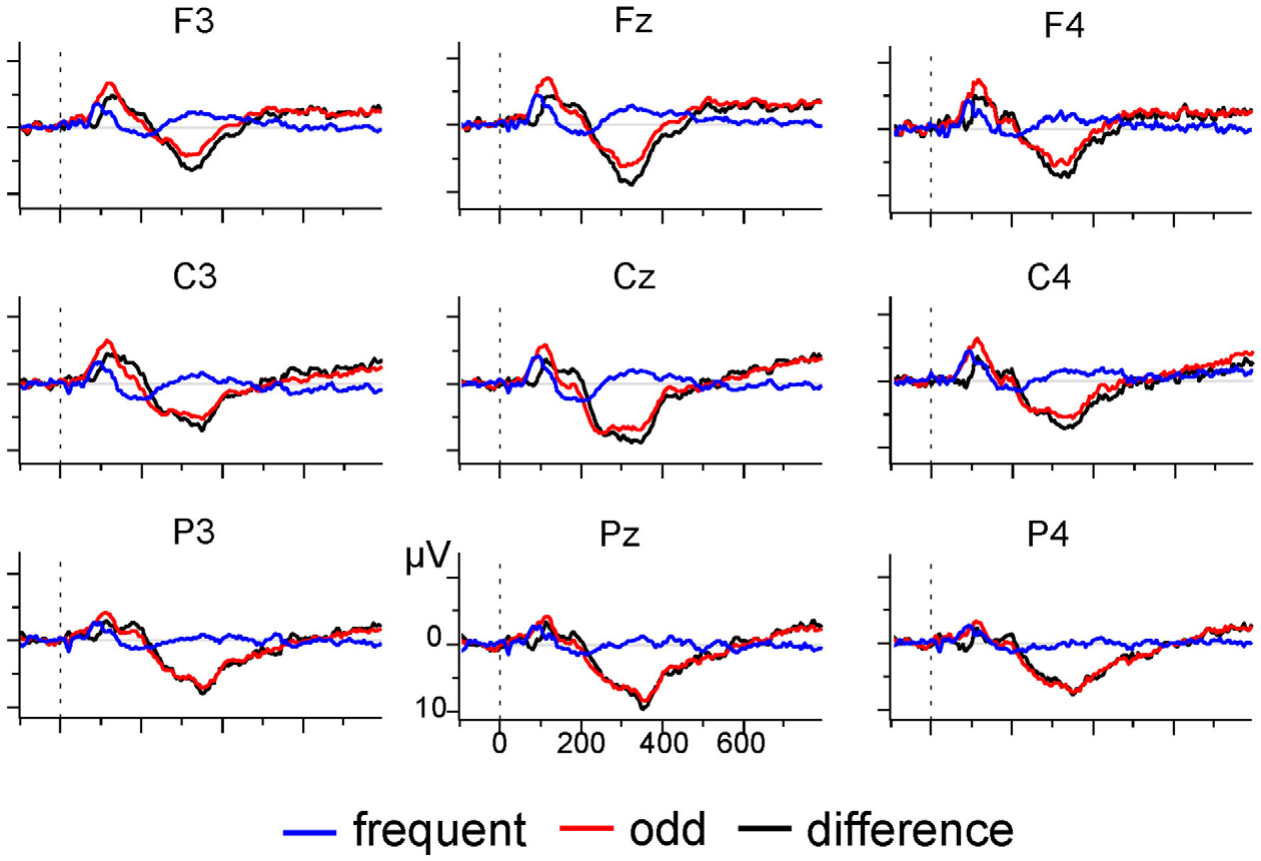
**Grand average of activation following odd and frequent tone trials (*N* = 14)**.

EEG was recorded with a sampling rate of 512 Hz using a 31-channel active electrodes cap (LADYbird, g.tec medical engineering, Schiedlberg, Austria; nose reference). Vertical and horizontal eye movement was recorded with two pairs of electrodes at the outer canthi and above and below one eye. Offline, data was bandpass (0.01–70 Hz) and notch (50 Hz) filtered, segmented into epochs of 850 ms, and aligned to the 100 ms pre-stimulus baseline. Ocular artifacts were corrected with a regression-based approach after which segments with absolute voltages exceeding 120 μ*V* were rejected as artifacts. Segments were re-referenced to linked-mastoids, and all odd tone trials and the preceding frequent tone trials selected for further analysis. Mean number of trials after artifact rejection was 52.93 (*SD* = 9.88) for each condition. Inspection of the grand average (see Figure 5) indicated the presence of a broad positive difference ERP (odd minus frequent tone trials) which was maximal at electrode Pz. Therefore, analysis was restricted to identifying a positivity at electrode Pz in the 250 ms long interval starting at 250 ms after stimulus onset (Polich, 2007). SNR estimates for these datasets were calculated on the basis of the sample correlation coefficient (Coppola et al., 1978, Equations 3–6, coefficient $\hat{\alpha }$_*R*_).

#### 2.4.3. Analysis

The *t*-CWT was hypothesized to be especially suited for the analysis of data with low SNRs. However, real EEG data from healthy participants only offers a limited range of SNRs. Therefore, analysis focused on EEG datasets obtained from healthy participants with degraded SNRs. First, datasets were split into “signal” and “noise” trials by calculating the “signal” as the single-subject difference ERP (activation in odd minus activation in frequent tone trials) and then calculating surrogate (Fell et al., 1996) “noise” trials by subtracting the signal from the single trials. Then, the signal's amplitude was reduced to achieve a desired SNR (ranging from −18 to −13 dB). Finally, the degraded signal and noise were recombined and subjected to further analysis. During the generation of datasets with degraded SNRs only those datasets in which the original SNR was above the to be simulated SNR were used (see **Table 3**). For example, a dataset with an original SNR of −12 dB would be used to generate degraded datasets ranging from −13 dB to −18 dB, while a dataset with an SNR of −14 dB would not be used to generate a dataset of −13 dB, as doing so would correspond to amplification instead of degradation. This method allowed us to analyze the performance of the *t*-CWT and the *t*_max_ test under several low SNRs while simultaneously maintaining properties of authentic EEG data (Fell et al., 1996). We hypothesized increased performance of the *t*-CWT at low SNRs. Differences between the number of identified datasets between the *t*-CWT and the *t*_max_ procedure were evaluated using a permutation test (1000 repetitions). Development of the *t*-CWT and *t*_max_ procedures was done in Python using the SciPy and NumPy libraries (Jones et al., 2001) and R (R Development Core Team, 2011) was used for statistical analysis.

## 3. Results

### 3.1. Results of simulation studies

Table 1 shows mean sensitivities and specificities for each analysis method and level of SNR. Procedures that do not correct for multiple testing [Simple peak detection and Peak detection (filtered)] show very high sensitivity but at the same time very high rates of false positives which exceed the nominal α-level of 0.05. In contrast, procedures based on the *t*_max_ randomization test do not show inflated false positive rates. The range-based peak detection procedure does not show α error inflation, however, sensitivity is lower as compared to the *t*_max_ (filtered) or the *t*-CWT procedure.

**Table 1:** **Sensitivity (SE) and 1-specificity (SP) by analysis method and SNR**.

**Analysis**	**Measure**	**SNR (dB)**					
		**−18**	**−17**	**−16**	**−15**	**−14**	**−13**
Simple peak detection	1-SP	0.9280	0.9270	0.9220	0.9340	0.9230	0.9230
Simple peak detection	SE	0.9870	0.9950	0.9990	1.0000	1.0000	1.0000
Peak detection (filtered)	1-SP	0.7920	0.7960	0.8110	0.8010	0.7860	0.8240
Peak detection (filtered)	SE	0.9950	1.0000	1.0000	1.0000	1.0000	1.0000
*t*_max_ (unfiltered)	1-SP	0.0590	0.0610	0.0440	0.0380	0.0500	0.0470
*t*_max_ (unfiltered)	SE	0.1910	0.2500	0.3470	0.4820	0.6520	0.7870
*t*_max_ (filtered)	1-SP	0.0490	0.0500	0.0660	0.0500	0.0470	0.0450
*t*_max_ (filtered)	SE	0.5800	0.7450	0.8620	0.9460	0.9880	0.9980
Rangebased peak detection (filtered)	1-SP	0.0120	0.0240	0.0120	0.0240	0.0230	0.0230
Rangebased peak detection (filtered)	SE	0.2860	0.3500	0.4580	0.5990	0.7210	0.8280
t-CWT	1-SP	0.0200	0.0220	0.0190	0.0150	0.0260	0.0180
t-CWT	SE	0.7460	0.8690	0.9490	0.9780	0.9940	1.0000

Figure 4 shows the mean *F*_1_ scores and associated 95% confidence intervals for each method studied. The non-overlapping confidence intervals for the *t*-CWT show that its *F*_1_ scores are significantly higher than those of the competing methods. From Figure 4 it also appears that the difference between the *t*-CWT and the *t*_max_ (filtered) procedure is smaller at higher levels of SNR. However, Table 2 confirms that the *t*-CWT achieves higher *F*_1_ scores than the *t*_max_ (filtered) procedure.

**Table 2:** **Results of the statistical comparison of the point estimates $\hat{F}$_1_(4, 7) between the *t*-CWT and the *t*_max_ (filtered) procedure by SNR**.

**SNR (dB)**		***$\hat{F}$*_1_scores**			**Negative *$\hat{F}$*_**1**_ scores**	
	***t*-CWT**	***t*_max_ (filtered)**	***p***	***t*-CWT**	***t*_max_ (filtered)**	***p***
−18	0.8448	0.7121	0.0000	0.8774	0.8022	0.0000
−17	0.9191	0.8301	0.0000	0.9275	0.8617	0.0000
−16	0.9644	0.8942	0.0000	0.9656	0.9015	0.0000
−15	0.9814	0.9479	0.0000	0.9816	0.9481	0.0000
−14	0.9842	0.9710	0.0026	0.9838	0.9700	0.0021
−13	0.9911	0.9770	0.0002	0.9909	0.9760	0.0001

### 3.2. Results of EEG analysis

The median $\hat{\alpha }$_*R*_ SNR for the difference in activation between odd and frequent tone trials in data recorded from healthy participants was −9.28 dB (*M* = −9.26, *SD* = 3.09), and 50% of participants had values between −8.99 and −1.87 dB. These estimates closely replicate previous findings on the distribution of SNRs obtained from healthy participants during auditory paradigms (Coppola et al., 1978, median = − 9.35 dB, 50% range: −7.17 to −1.31 dB).

Using the *t*_max_ randomization test after 4th-order Butterworth band pass filtering (0.1–20 Hz) indicated that 6 out of 14 (43%) participants showed a significant difference in activation between odd and frequent tone trials at Pz. In contrast, the *t*-CWT could detect a significant positivity in two additional participants (total: 8 of 14, 57%).

Table 3 shows the results of our main analysis. At high SNRs the *t*-CWT and the *t*_max_ show increasingly high agreement, but at lower levels of SNR the *t*-CWT identified more significant differences than the *t*_max_ test. In total, the *t*-CWT identified significantly (*p* = 0.001) more datasets (*n* = 39) than the *t*_max_ test (*n* = 14).

**Table 3:** **Percentage of degraded datasets (~ participants) with a significant positivity in the P300-time range**.

	**SNR (dB)**					
	**−18**	**−17**	**−16**	**−15**	**−14**	**−13**
*n*^a^	13	13	12	11	9	9
*t*-CWT	15.00	31.00	67.00	82.00	89.00	89.00
*t*_max_	0.00	0.00	17.00	27.00	33.00	67.00

a The number of participants whose SNR was higher than that required for simulation (see Section 2.4.3).

## 4. Discussion

In this study, we evaluated the performance of a variant of the Studentized Continuous Wavelet Transform (*t*-CWT). Earlier studies based on data from healthy participants suggested favorable performance, however, specificity and performance under different signal-to-noise ratios were not evaluated. Using simulated EEG datasets in which a signal was either present or absent at six levels of low SNR allowed us to systematically analyze the performance of a variety of EEG signal detection methods and compare them to the *t*-CWT. Our results show that for peak detection procedures that do not control for multiple comparisons, false positive rates (greatly) exceed the nominal α level. In contrast, procedures using *t*_max_ randomization tests effectively control the false positive rate. The *t*-CWT showed superior performance compared to all other examined methods. Analysis of EEG data obtained from healthy participants while listening to a two-tone auditory oddball paradigm showed that the *t*-CWT identified a significant difference ERP in the P300-time range in more participants than the *t*_max_ test. Further, analysis of surrogate EEG data confirmed that the *t*-CWT is particularly sensitive at low SNRs.

Filtering has long been used to increase SNRs and much effort has been spent on identifying optimal filtering procedures for ERP detection (e.g., Kalyakin et al., 2007 for the MMN, and Farwell et al., 1993 for the P300). However, these approaches rely on using just one optimal filter, thereby running the risk of attenuating ERPs, which do not match the filter specifications. In contrast, the wavelet approach of the *t*-CWT can be thought of simultaneously applying a multitude of filters, thereby increasing the chance of identifying an optimum. Thus, the *t*-CWT also allows for the detection of several ERPs simultaneously, e.g., detecting the N100-(P200) complex and a P300 in an oddball paradigm.

However, this conceptual superiority comes at increased computational costs, as the time required for the *t*_max_ randomization tests increases with the number of wavelet coefficients, and the number of randomizations. Other methods to control for multiple comparisons exist, e.g., the variants of the false discovery rate (FDR; Benjamini and Hochberg, 1995; Benjamini and Yekutieli, 2001; Benjamini et al., 2006) and have also been applied to wavelet coefficients (Abramovich and Benjamini, 1995). These were not implemented as they all entail stronger assumption of the underlying data structure than the *t*_max_ randomization test, which only assumes symmetric distribution around zero under *H*_0_. However, closer examination of the performance of the *t*-CWT when using FDR might still be worthwhile since these procedures work much faster than randomization tests.

EEG analysis methods are complex, results sometimes only depend on subtle differences in the preprocessing procedures (e.g., VanRullen, 2011; Acunzo et al., 2012) or statistical analysis (e.g., Cruse et al., 2011, 2013; Goldfine et al., 2013), and it may not always be easy to decide upon the most appropriate method. However, the use of simulated data offers the possibility of systematically varying the data's properties a particular analysis method is designed to detect. It, thus, offers a controlled testing environment that may help to tailor an analysis for a particular problem. Nevertheless, simulations can only approximate real life data and results are strongly influenced by the underlying assumptions. For example, although our analysis of real EEG data confirmed results obtained during simulation, it appears that sensitivities of both *t*-CWT and *t*_max_ test are overestimated during simulation (see sensitivities in Table 1) in comparison to real EEG data (Table 3). We speculate the reason for this to be that the assumptions made during simulation, i.e., a highly localized peak embedded in Gaussian white noise, while statistically convenient, are less than perfect approximations to real EEG data. Importantly, however, the most important finding from our simulation study – high sensitivity of the *t*-CWT– was validated by the analysis of EEG data from healthy participants.

The assessment of ERPs promises to be a valuable tool in determining residual cognitive functions in patients with DOC. However, a variety of factors may lead to reduced signal-to-noise ratios in EEG obtained from these patients, making reliable assessment difficult. At the same time, depending on the results of the assessment, consequences may be far reaching (Laureys et al., 2006; Eisenberg, 2008). Using simulated ERPs at six low levels of SNR we have shown that the *t*-CWT was superior to a variety of other procedures in terms of sensitivity, specificity, positive (~*F*_1_ scores), and negative predictive values (~ negative *F*_1_ scores).

While the development of the *t*-CWT was prompted by a desire to evaluate ERPs in DOC patients, the method can be applied in other scenarios, as it can be used whenever detection of ERPs in single subjects is necessary. However, it should be noted that its increased sensitivity might not be noticeable provided high SNRs. Thus, the *t*-CWT may be best for the assessment of weak ERPs. Finally, although we have used a real wavelet in our study, the *t*-CWT can be easily extended to include complex wavelets, thus, allowing for the analysis of non-phase-locked activity, or to compensate for latency jitter.

### Conflict of interest statement

The authors declare that the research was conducted in the absence of any commercial or financial relationships that could be construed as a potential conflict of interest.

## Footnotes

^1^Note, the use of σ = 1/4 instead of the standard σ = 1 in (2). In the standard definition, σ corresponds to half the width between the zeroes of the Mexican Hat, while in our definition σ corresponds to the distance between the minima. This was done for convenience, as, thus defined, the scale parameter corresponds to the approximate wavelength of the Mexican Hat (inverse frequency). In ERP applications, scales can then be interpreted as the approximate durations of components.
